# Genetic Regulation of Vessel Morphology in *Populus*

**DOI:** 10.3389/fpls.2021.705596

**Published:** 2021-08-23

**Authors:** F. Daniela Rodriguez-Zaccaro, Isabelle M. Henry, Andrew Groover

**Affiliations:** ^1^US Forest Service, Pacific Southwest Research Station, Davis, CA, United States; ^2^Department of Plant Biology, University of California, Davis, Davis, CA, United States

**Keywords:** forest tree, drought, wood formation cambium activity, genomics, forest tree growth

## Abstract

During secondary growth, forest trees can modify the anatomy of the wood produced by the vascular cambium in response to environmental conditions. Notably, the trees of the model angiosperm genus, *Populus*, reduce the risk of cavitation and hydraulic failure under water stress by producing water-conducting vessel elements with narrow lumens, which are more numerous and more interconnected with each other. Here, we determined the genetic architecture of vessel traits affecting hydraulic physiology and resilience to water stress. Vessel traits were measured for clonally replicated genotypes of a unique *Populus deltoides* x *nigra* population carrying genomically defined insertions and deletions that create gene dosage variation. We found significant phenotypic variation for all traits measured (mean vessel diameter, height-corrected mean vessel diameter, vessel frequency, height-corrected vessel frequency, vessel grouping index, and mean vessel circularity), and that all traits were under genetic control and showed moderate heritability values, ranging from 0.32 to 0.53. Whole-genome scans of correlations between gene dosage and phenotypic traits identified quantitative trait loci for tree height, mean vessel diameter, height-corrected mean vessel diameter, height-corrected vessel frequency, and vessel grouping index. Our results demonstrate that vessel traits affecting hydraulic physiology are under genetic control, and both pleiotropic and trait-specific quantitative trait loci are found for these traits.

## Introduction

Wood (secondary xylem) is the water conducting tissue of tree stems, and the anatomical features of wood can have profound effects on water transport and vulnerability to hydraulic failure during water stress (Rodriguez-Zaccaro and Groover, 2019). Vessel elements are the primary water conducting cells in most angiosperms including trees of the model genus *Populus*. Vessel differentiation starts with the commitment of terminal cell fate and cessation of cell division, followed by cell expansion, construction of a rigid secondary cell wall, and programmed cell death to produce water conducting cell corpse (Groover and Jones, 1999; Turner et al., 2007). In *Populus*, stems undergoing secondary growth, the vessel elements differentiate from the xylem mother cells derived from the fusiform initials of the vascular cambium and join end-on-end to create longer water conducting tubes termed as vessels (Larson, 1994; Sperry et al., 2006). Pits between adjacent vessel elements and between vessel elements and other cells, such as ray parenchyma, provide additional routes for water to pass both between and out of vessel elements. Importantly, secondary growth can be altered to produce wood anatomy and vessel morphologies that mitigate water stress during drought or maximize water conduction for fast growth under permissive conditions (Tyree, 1989; Tyree and Sperry, 1989; Tyree and Zimmermann, 2002). To what degree these changes reflect the passive or indirect responses of development to environmental conditions, as opposed to genetically regulated responses, remains unclear.

Together, the anatomical properties of vessels have a profound effect on water conduction physiology (Rodriguez-Zaccaro and Groover, 2019). Vessel lumen diameter directly affects the rate of water transport through secondary xylem with wider vessels contributing to greater water transport efficiency. This can be partially explained through the Hagen-Poiseuille law, modeling the volumetric flow rate of a liquid moving through a tube as being proportional to the fourth power of the radius of the tube (Venturas et al., 2017). Consequently, a small increase in vessel width can produce a large increase in water flow and allow for greater photosynthesis and growth rates (Brodribb and Feild, 2000). Larger vessel diameters, however, have been associated with greater vulnerability to cavitation due to drought and other abiotic stressors (Gleason et al., 2016; Hacke et al., 2016). Cavitation results in the formation of air-pockets within the xylem network, which can lead to lethal hydraulic failure (Tyree, 1989). Vessel frequency (VF), which is the number of vessels within a given area of xylem, and vessel grouping index (VGI), a measure of vessel clustering and interconnectivity, can also affect hydraulic function. A greater VF can increase water transport efficiency and buffer the xylem network from hydraulic failure by allowing for a larger fraction of smaller diameter vessels that remain functional under water stress compared to lower VF xylem (Baas et al., 1983; Villar-Salvador et al., 1997). Similarly, a greater VGI can lead to increased hydraulic efficiency and mitigate the effects of cavitation by providing xylem sap alternate routes to bypass embolized vessels (Carlquist, 1984; Lens et al., 2011). Greater VGI, however, has also been related to increased vulnerability to cavitation due to a likely increase in the probability of embolism spread in a more interconnected xylem network (Loepfe et al., 2007). A trait that has not been well studied in the context of hydraulic function is vessel shape or cross-sectional circularity. According to microchannel fluid mechanics, a more circular conduit can transport fluids more efficiently than conduits with more irregular shapes (White, 2011). Vessel circularity has also been related to safety from water stress with more circular vessels able to withstand large negative pressures within the xylem network without imploding and disrupting water transport (Cochard et al., 2004). Non-lumen fraction (NF) is the proportion of xylem area that is not made up of vessel lumen area. This trait has been previously positively related to wood mechanical strength and density (Searson et al., 2004; Zanne et al., 2010), an important trait that helps to determine commercial wood quality.

Several stem and wood anatomical traits have been shown to be correlated to each other (von Arx et al., 2013; Hajek et al., 2014) although cause and effect for these correlations are not known. For example, mean vessel diameter (MVD) and VF are often negatively correlated (Chauhan et al., 1999; Sellin et al., 2008). Some studies have suggested that this correlation is the result of a trade-off between hydraulic conductivity and mechanical support in stems (Wagner et al., 1998; Barbour and Whitehead, 2003); a positive correlation leading to larger and more numerous vessels could result in mechanically weaker stems that are selected against. MVD and VF are also known to predictably scale with tree height (TH); larger trees tend to have wider and less numerous vessels compared to smaller trees at the same sampling height (Olson et al., 2014). Some hydraulic optimality models suggest that vessel width and quantity are influenced by height. The Hagen-Poiseuille law predicts that longer conduits are more resistant to flow than shorter conduits of equal diameter. Wider vessels, then, are thought to compensate for the decrease in water transport efficiency that would otherwise result from the longer conductive pathways necessarily present in taller trees (Olson and Rosell, 2013). However, the correlation between tree height and vessel traits is not perfect, and it is unclear to what degree the observed variation in vessel traits could be the result of genetic regulation independent from tree size.

The regulation of cell expansion and final diameter in vessel elements likely involves the regulation of cell turgor and cell wall expansion. The experimental manipulation of potassium, a primary osmoticum of plant cells likely involved in cell turgor regulation, can be used to alter the diameter of differentiating vessel elements (Cutter and Morphey, 1978). However, there is less evidence that the manipulation of secondary cell wall formation can have similar effects. For example, well-characterized *irregular xylem* (*irx*) mutants with physically compromised vessels with crumpled cell walls are not significantly defective in terms of cell diameter (Turner and Somerville, 1997). Whether turgor regulation, cell wall regulation, or other unknown factors, such as the timing of transition from cell division to cell expansion, are the most critical factors that influencing the final cell diameter and morphology remains uncertain. One reason for this knowledge gap is that experimental systems to investigate the genetic and genomic properties of vessel element morphology within relevant species, including forest trees, have been historically lacking.

We previously developed *Populus* germplasm with the goal of performing functional genomic experiments directly in a forest tree species with extensive secondary growth and wood formation. To create this resource, pollen from a *Populus nigra* male tree was irradiated to create chromosomal breaks prior to crossing with two *Populus deltoides* females to produce a large F1 hybrid population (Henry et al., 2015). The insertions and deletions in each F1 individual create structural variation and associated gene dosage variation in the affected regions. Insertions and deletions were genomically mapped for each F1 individual, allowing genome-wide surveys linking gene dosage variation with phenotypes to identify dosage quantitative trait locus (dQTL; Bastiaanse et al., 2019, 2021). The genetic architecture of multiple biomass and phenology-related traits were previously dissected using this resource, including the identification of dQTL for multiple traits, and demonstrated that gene dosage is a major source of phenotypic variation in this population (Bastiaanse et al., 2019, 2021). These results are relevant to natural genetic variation in *Populus*, where, similar to other plant species that have been surveyed, structural and gene dosage variation is prevalent (Pinosio et al., 2016; Zhang et al., 2019).

Here, we used the same poplar irradiation hybrid germplasm to estimate the genetic architecture of prominent vessel element and stem traits including MVD, VF, VGI, mean vessel circularity (MVC), tree height (TH), non-lumen fraction (NF), and bark thickness (BT). We report correlations among all traits measured including tree height and vessel traits. All traits showed modest heritabilities, indicating that there is a significant genetic component underlying the observed phenotypic variation for vessel traits. Additionally, dQTL are reported for MVD, tree height-corrected mean vessel diameter (cMVD), tree height-corrected vessel frequency (cVF), and VGI. We provide evidence of dQTL commonly shared by correlated traits, as well as trait-specific dQTL, suggesting that independent regulatory factors exist for both correlated and uncorrelated traits. Together, our results show that vessel traits are under genetic regulation and are not simply a passive consequence of tree height, and that system genetic approaches could now be used to further dissect these traits and identify candidate genes using this same genomics resource.

## Materials and Methods

### Plant Materials

A subset of 201 poplar hybrid genotypes was included from a larger dosage mutant pedigree developed and described by Henry et al. (2015). Briefly, the pedigree was produced by crossing two female *P. deltoides* with gamma-irradiated pollen of a male *P. nigra*. The F1 hybrids were then completely sequenced using Illumina short reads. Relative sequencing read coverage values were used to detect the insertions and deletions (indel mutations) across F1 hybrids that together cover the entire poplar genome multiple times. The pedigree consists of nearly 800 replicated lines maintained in an outdoor plantation at the US Forest Service Institute of Forest Genetics in Placerville, CA.

All lines within the subset were clonally propagated through stem cuttings from the field in multiple replicates (ramets) during the spring of 2018. Cuttings were planted with rooting hormone (Bontone) in individual 2.83-L pots filled with horticultural soil (Sungro Sunshine Mix #4) and fertilizer (Osmocote, approximately 14 g/kg soil) inside a greenhouse at the IFG. After a 2-month growth period, three healthy clonal replicates per line were randomly selected to include in a randomized complete block design. Plants were grown in a lathe house for 3 months, harvested, and moved inside a greenhouse to coppice and grown for a second 3-month period before harvesting a second time. Both crops were kept under well-watered conditions using a drip-irrigation system and monitored stem height and diameter growth until the end of the growing season in the fall of 2018. Greenhouse trees were kept at a near constant temperature of 23°C. All analyses and results involve the latter greenhouse-grown crop. A 6–8-cm woody stem segment was harvested from each tree at a fixed height of 10 cm from the point of emergence from the original cutting. Stems were immediately stored in 60% ethyl alcohol solution in 50 ml Falcon tubes.

### Histology

Stem internodes were cut in 40 μm thick cross-sections with a sliding microtome (Spencer Lens) or a vibratome (Vibratome Series 1000). Sections were stained with and mounted in a mixture of phloroglucinol and Astra Blue, which stain lignin and cellulose, respectively. Sections were then photographed at ×5 or ×10 magnification under a microscope with a digital camera with standardized settings (Leica Microsystems). A 100-μm scale bar was included in each image, adjusted for specific magnification. Lastly, a high-quality micrograph of each replicate within a line was selected for image analysis.

### Image Analysis

Stem cross-section micrographs were processed using Fiji ImageJ software (v2) to obtain wood anatomical trait data. Images were spatially calibrated using the known scale bar length to determine the number of pixels per micrometer. Bark thickness was calculated as the average distance between the cambial zone and the outer cork in unprocessed images. All non-xylem areas (bark and pith) were then manually cut out of the image before converting to grayscale. Images were divided into a “foreground” consisting of vessel lumens and a “background” made up of non-vessel lumen area by setting a standardized pixel thresholding value. Vessel lumen area and circularity values were obtained directly through the Analyze Particles tool with equivalent circle vessel diameters calculated from vessel lumen areas (Scholz et al., 2013). A MVD and MVC value was calculated for each image. A vessel frequency value was calculated for each image by dividing the number of vessels (obtained through the Analyze Particles tool) by the total xylem area (mm^2^). NF was calculated by multiplying the mean vessel area by vessel frequency (Scholz et al., 2013). A VGI value was obtained for each image by dividing the number of vessels by the number of vessel groups. A vessel group consists of anything from a solitary vessel to any number of clustered vessels with secondary walls that are in contact (Carlquist, 2001). Vessel groups were counted manually using the Multi-point tool.

### Height Corrected Traits

Because tree height is correlated with vessel diameter (Olson and Rosell, 2013), cMVD, and cVF values were calculated for each tree to assess the portion of trait variation that cannot be explained by height. The log10 of the final tree height at harvest was plotted against the log10 of each trait. The equation describing the linear regression of each plot was used to obtain expected trait values predicted from tree height. Expected values were subtracted from observed trait values to obtain residual values that were used as height-corrected trait data. Lines with residual values near 0 were considered to have vessel trait values expected for their height. Lines with significantly higher residual values for MVD, for example, were considered to have unusually wide vessels for their height.

### Estimating Broad-Sense Heritability

The broad-sense heritability (*H*^2^) of each trait was estimated using the repeatability function in the CRAN heritability R package (version 1.3) developed by Kruijer et al. (2015). Repeatability, or intraclass correlation, is considered as the broad-sense heritability and was calculated by dividing the total genetic variance by the total phenotypic variance of a trait. The total genetic variance was estimated by subtracting the mean sums of squares for genotype and residual error obtained from an analysis of variance and dividing the results by the number of clonal replicates in each line. The total phenotypic variance was calculated by adding the total genetic variance to the mean sums of squares of the residual error (Kruijer et al., 2015).

### Statistical Analysis

Trait data were transformed, when appropriate, through Box-Cox power transformations. Pearson’s correlation tests were run to determine the relationship between all trait combinations. An ANOVA-based analysis was performed on each trait in base R, with genotype and final tree height at harvest as independent factors in each model, with the exception of height-corrected traits, which involved only a genotype factor. Tree height was also treated as a dependent variable in a separate model with genotype as the sole independent factor. Each analysis was followed by a Tukey’s honest significance *post hoc* test.

Dosage-dependent quantitative trait locus analyses (dQTL) were performed on all traits. The start and end sites of all the indels of the genotypes included in the analysis were used to create the boundaries of genomic bins as previously described (Bastiaanse et al., 2019, 2021). A relative gene dosage score was calculated for each genotype at each genomic bin by dividing the gene dosage at the bin by the background ploidy of the particular line. A Kendall’s tau coefficient was calculated at each bin to test for statistical dependence between the relative dosage score (RDS) and the phenotypic trait data (Schaeffer and Levitt, 1956). Most indels encompassed more than one chromosomal bin, and contiguous bins were thus correlated, as previously described for this population (Bastiaanse et al., 2021). Consequently, the resulting values of *p* were adjusted through a modified Bonferroni correction for multiple testing, in which the values of *p* were multiplied by the number of independent chromosomal bins (Bastiaanse et al., 2021). The number of independent bins was obtained through a dissimilarity matrix by calculating pairwise correlation coefficients between the relative dosage ratios of bins across all genotypes. These correlation coefficients were grouped using a hierarchical clustering method and individual branches were combined using a cutoff value of 0.7 (Bastiaanse et al., 2021). The original 469 genomic bins were merged into 40 independent bins through this method. The adjusted values of *p* below a threshold of 0.05 were considered as significant and signaled putative dQTL associated with a trait. The adjusted *R*-squared of the linear regression model fitting genomic bins was used to estimate the percentage of trait variance explained by dQTL.

## Results

**Figure 1:**
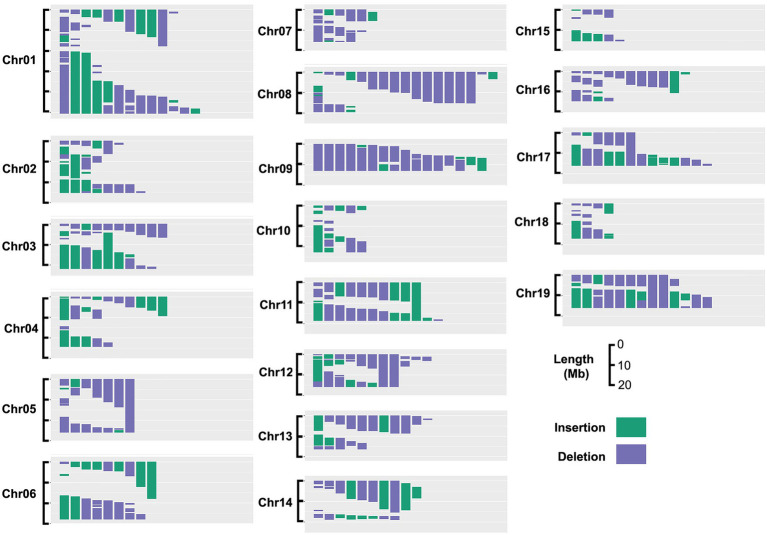
Insertion and deletion (indel) coverage across the poplar genome for genotypes included in this study. Based on the indel size and location in the 201 genotypes chosen for this study, 95% of the genome is covered by at least one indel.

Here, we used a subset of a previously described population of *P. deltoides* x *nigra* carrying genomically mapped indels (Henry et al., 2015) to investigate the effects of gene dosage variation on stem anatomical and vessel traits. Of the 201 genotypes included in the analysis, 173 carried insertions and/or deletions, with the remaining lines included as non-indel controls. Genomic bins were defined by the breakpoints of indels across all lines included in this experiment (see section “Materials and Methods”), which represented 469 genomic bins covering 91.5% of the genome, as shown in Figure 1. Genomic bin size ranged from 0.01 to 5.2 Mb with an average bin size of 0.78 Mb. Lesion coverage varied substantially between chromosomes, ranging from 1 to 16 indels per genomic bin, with a genome-wide average of six indels per bin.

**Figure 2:**
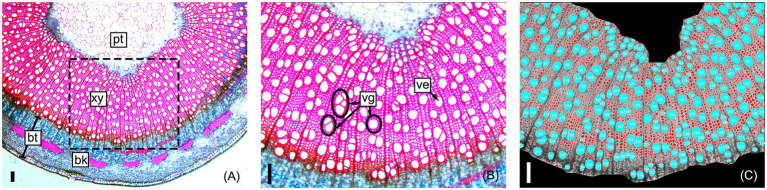
Representative sample of a poplar stem cross-section used to obtain stem and vessel anatomical trait data. Section micrographs include the pith (pt), secondary xylem (xy), and bark (bk); bark thickness (bt) is measured before image processing **(A)**. Vessel elements (ve) and vessel group (vg) examples are shown within a close-up of the sampled section **(B)**; vessel groups are single vessels or any number of clustered vessels with secondary cell walls that are in contact. Image J software (v2) was used to measure xylem area, automatically count vessels, and measure vessel areas and circularities (see section “Materials and Methods”) within the secondary xylem **(C)**. All scale bars are 100 μm.

Clonal replicates of all genotypes were grown and measured under permissive greenhouse conditions, prior to destructive sampling and measuring wood anatomical and vessel traits (see section “Materials and Methods”). For each tree, a basal stem cross section was stained, digitally imaged, and analyzed to extract anatomical trait data as illustrated in Figure 2. Traits directly measured or calculated are summarized in Table 1 and include tree height (TH), MVD, vessel frequency (VF), VGI, MVC, non-lumen fraction (NF), and bark thickness (BT). Some hydraulic optimality models suggest that vessel width and quantity are a consequence of organ size (Olson and Rosell, 2013). Both MVD and VF are known to be highly correlated to tree height, where larger trees have wider and less numerous vessels compared to smaller trees at the same sampling height (Olson et al., 2014). To assess the portion of trait variation not explained by height, we evaluated correlations among raw anatomical and vessel trait data and tree height and included height-corrected adjuncts for traits with significant correlations (see section “Materials and Methods”). Two traits were corrected this way: cMVD and cVF (Table 1).

**Table 1:** The means, standard deviations (SD), and ranges of phenotypic traits measured in a 201 genotype poplar pedigree.

Trait	Abbr.	Units	Mean	SD	Range
Tree height	TH	cm	64	32	5–168
Mean vessel diameter	MVD	μm	26	4.0	14.0–34.9
Height-corrected MVD	cMVD	Unitless	0.0001	0.043	−0.136–0.123
Vessel frequency	VF	vessels/mm^2^	365	166	165–1,000
Height-corrected VF	cVF	Unitless	−0.0004	0.084	0.341–0.282
Vessel grouping index	VGI	vessel/vessel group	1.47	0.1	1.17–1.79
Mean vessel circularity	MVC	Unitless	0.78	0.03	0.68–0.87
Non-lumen fraction	NF	Unitless	0.8	0.04	0.66–0.91
Bark thickness	BT	μm	608	349	169–779

### Pedigree Trait Distributions

**Figure 3:**
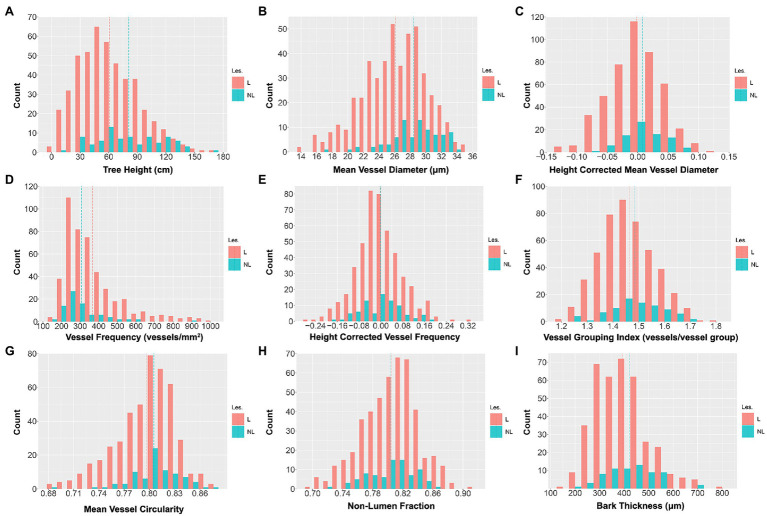
Untransformed wood anatomical trait and stem trait data distributions across the 201 genotypes included in the study. Values are for three clonal replicates per genotype. The red bars represent indel genotypes (173), and the blue bars represent non-lesion genotypes (28). Traits include tree height (TH; **A**), mean vessel diameter (MVD; **B**), height-corrected mean vessel diameter (cMVD; **C**), vessel frequency (VF; **D**), height-corrected vessel frequency (cVF; **E**), vessel grouping index (VGI; **F**), mean vessel circularity (MVC; **G**), non-lumen fraction (NF; **H**), and bark thickness (BT; **I**). The red dotted lines represent the trait means of indel lines, and the blue dotted lines represent the trait means of non-indel lines.

Distributions of raw trait data are shown in Figure 3; trait distribution means, standard deviations, and ranges are summarized in Table 1. All traits showed continuous variation, consistent with multigenic variation expected for classical quantitative traits. The final tree height at harvest varied substantially within the population, ranging from 5 to 168 cm, with a mean height of 64 and a standard deviation of 32 cm (Table 1; Figure 3A). MVD within the 201 genotype pedigree subset ranged from 14 to 36 μm (Figure 3B), with an average of 26 and a standard deviation of 4 μm. VF distribution was strongly skewed right (Figure 3D) and ranged from 165 to 1,000 vessels/mm^2^ of xylem, with an average of 365 and a standard deviation of 166 vessels/mm^2^. VGI had a narrow distribution (Figure 3F) that ranged from 1.17 to 1.79 vessels/vessel group with a mean of 1.47 and a standard deviation of 0.1 vessels/vessel group. MVC had a narrow distribution (Figure 3G) that ranged from 0.68 to 0.87, with an average of 0.78 and a standard deviation of 0.03. NF distribution was narrow (Figure 3H) and ranged from 0.66 to 0.91 with a mean of 0.80 and a standard deviation of 0.04.

In comparison with non-indel control genotypes, indel genotypes extended the observed variation and defined the extremes of each phenotype as shown in Figure 3 and Table 2. Thus, indels generated phenotypic variation beyond the variation caused by allelic segregation. Consistent with previous findings (Bastiaanse et al., 2019), as a group lines with lesions had an overall negative effect on growth, as illustrated here by significantly lower tree heights for the lesion group (Table 2). Additional traits with significantly smaller means in the lesion line group included MVD and BT, while lesion lines had a larger mean for VF (Table 2). Thus, MVD, BT, and VF behave as growth-related traits with regards to response to lesion-induced dosage variation.

**Table 2:** The means, standard deviations (SD), and ranges of phenotypic traits measured in 201 genotype poplar pedigree across non-lesion (NL Lines) and lesion genotypes (L Lines).

Trait	NL Lines			L Lines		
	Mean	SD	Range	Mean	SD	Range
TH	80.9	34.6	9–166	60.8^**^	30.8	5–168
MVD	28.4	3.3	16.6–34.3	26.1^**^	4.0	14.0–34.9
cMVD	0.008	0.032	−0.064–0.082	−0.004	0.044	−0.136–0.123
VF	307	119	165–902	375^*^	171	168–1,000
cVF	0	0.074	−0.179–0.185	0	0.086	−0.282–0.341
VGI	1.48	0.1	1.25–1.69	1.46	0.1	1.18–1.79
MVC	0.81	0.030	0.69–0.87	0.80	0.035	0.68–0.87
NF	0.80	0.031	0.73–0.87	0.80	0.037	0.66–0.91
BT	421	99	221–690	392	105	169–779

* Means different at ≤ 0.05.

** Means different at ≤ 0.01.

### Genotype Effect and Broad-Sense Heritability

**Figure 4:**
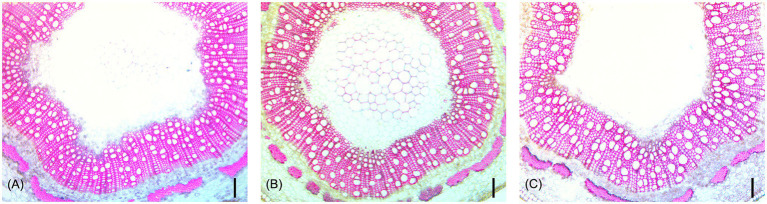
Representative stem cross-sections of three hybrid poplar genotypes that did not differ significantly in tree height (~30 cm), but show significant differences in vessel diameters. Genotype XXX_100_71 **(A)** had a MVD of 20 μm, XXX_100_70 **(B)** had a MVD of 25 μm, and XXX_100_93 **(C)** had a MVD of 29 μm. All scale bars are 100 μm.

ANOVA tests showed that genotype had a significant effect on all measured traits (Table 3). Both genotype (*p* < 0.0001) and tree height (*p* < 0.0001) had a statistically significant effect on MVD. There was a significant interaction between genotype and tree height (*p* < 0.0001), indicating a departure from what would be expected if MVD was directly attributed simply to tree height. Genotype (*p* < 0.0001) and tree height (*p* < 0.0001) had significant effects on VF, but there was no significant interaction between genotype and tree height (*p* = 0.0597). Genotype also had a significant effect on cMVD (*p* < 0.0001) and cVF (*p* < 0.0001), indicating significant genetic variance for these traits not directly attributable to tree size. An example of genotypes that significantly deviate from expected MVD based on tree height are shown in Figure 4. Genotype (*p* < 0.0001), but not tree height (*p* = 0.193), explained significant portions of VGI phenotypic variation. Genotype (*p* < 0.0001), but not tree height (*p* = 0.186), had a significant effect on MVC. Both genotype (*p* < 0.0001) and tree height (*p* < 0.0001) had a significant effect on NF.

**Table 3:** ANOVA test results and broad-sense heritabilities (*H*^2^) of phenotypic traits in a 201 genotype pedigree.

Trait	Line (*p*)	TH (*p*)	Line*TH (*p*)	*H* ^2^	95% CI
TH	<2e-16	–	–	0.45	±0.09
MVD	<2e-16	<2e-16	1.16E-06	0.49	±0.09
cMVD	<2e-16	–	–	0.44	±0.09
VF	<2e-16	<2e-16	0.0597	0.45	±0.09
cVF	9.93E-12	–	–	0.32	±0.09
VGI	1.38E-15	0.193	0.235	0.47	±0.09
MVC	<2e-16	0.186	0.393	0.51	±0.09
NF	<2e-16	4.70E-07	0.0799	0.5	±0.08
BT	5.45E-11	<2e-16	0.531	0.37	±0.09

The values of *p* were obtained from two-way ANOVA tests for each trait, with genotype (Line) and tree height (TH) as independent factors. TH, cMVD, and cVF were tested as dependent variables with line as the sole independent factor. *H*^2^ was estimated through intraclass correlation, with 95% confidence intervals for each heritability estimate shown.

Broad-sense heritabilities (repeatabilities) were calculated for all traits and are shown in Table 3. All wood and stem traits were moderately heritable. cVF had the lowest broad-sense heritability estimate among all traits (*H*^2^ = 0.32) with confidence intervals ranging from 0.22 to 0.41. MVC had the highest broad-sense heritability estimate (*H*^2^ = 0.51), with confidence intervals ranging from 0.42 to 0.59. As a reference to the estimation of vessel and stem anatomical trait heritabilities, tree height had a heritability of 0.45 in this study. All anatomical traits showed significant heritabilities suggest that the observed phenotypic variation for each trait has a substantial genetic basis. Together with the continuous phenotypic trait distributions, these results are consistent with the traits under study being quantitative traits influenced by multiple genes.

### Pearson’s Correlation Tests Between Traits

**Figure 5:**
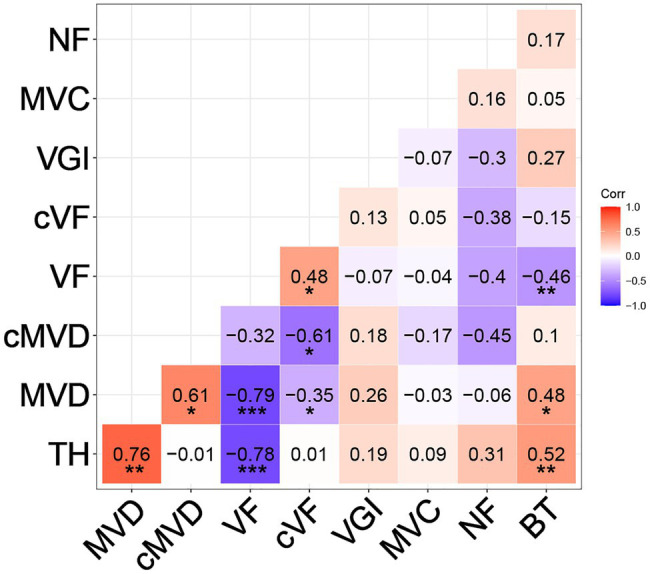
Pearson correlation coefficients between different wood and stem traits. Traits include tree height at harvest (TH), MVD, cMVD, VF, cVF, VGI, MVC, non-lumen fraction (NF), and bark thickness (BT). Asterisks indicate significant correlations between traits (^*^p ≤ 0.05; ^**^p ≤ 0.01; ^***^p ≤ 0.001).

Correlations among all traits were calculated (see section “Materials and Methods”) and are summarized in Figure 5. The final tree height at harvest was strongly positively correlated with MVD (*R* = 0.76, *p* < 0.01) and strongly negatively correlated with VF (*R* = −0.78, *p* < 0.001), similar to previous findings (Olson et al., 2014). Even after correcting for tree height, there was a negative correlation between cMVD and cVF (*R* = −0.61, *p* < 0.05), indicating potential interdependence of the size and frequency of vessel elements in wood that is independent of organ size. There was a weaker, but significant positive correlation between tree height and BT (*R* = 0.52, *p* < 0.01). VGI, MVC, and NF, however, were not significantly correlated to tree height or to any other trait, suggesting that these traits might be independently regulated from each other. MVD and VF were strongly negatively correlated (*R* = −0.79, *p* < 0.001), as previously reported (Olson et al., 2014), and suggest either common regulation or direct influence of one trait on the other. BT was moderately but significantly positively correlated to MVD (*R* = 0.48, *p* < 0.05) and negatively correlated to VF (*R* = −0.46, *p* < 0.01). BT could be considered as a proxy for radial growth, and thus correlated with greater MVD and lower VF as expected for faster growing and larger trees.

### Dosage-Dependent QTL Analyses

We used our previously described approach (Bastiaanse et al., 2019, 2021) for correlating gene dosage at genomic bins with phenotypes, and mapping dosage QTL for all stem and anatomical traits (see section “Materials and Methods”). Briefly, genomic bins were established based on indel breakpoints across all lines (genotypes). For each bin within each line, RDS was defined as 0.5 for deletions, 1.5 for insertions, and 1 for normal diploid dosage. Correlations among relative dosage at each bin and each phenotype was then assessed across all lines genome-wide to identify dQTL. Adjacent bins were not independent and were frequently spanned by indels (Figure 1); thus values of *p* were adjusted for multiple testing based on the effective number of uncorrelated bins (see section “Materials and Methods”).

**Figure 6:**
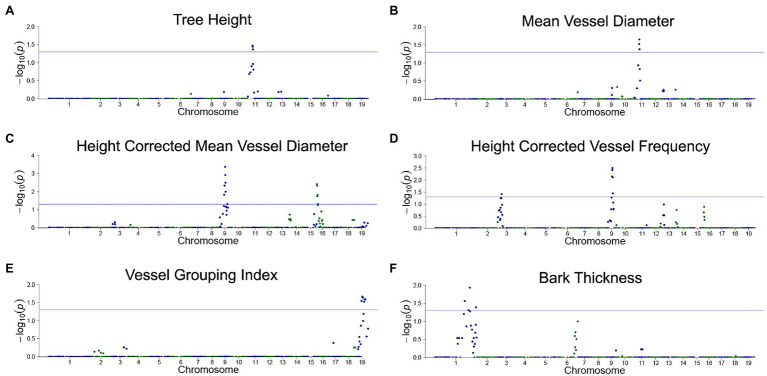
Dosage-dependent quantitative trait loci (dQTL) of traits. Traits with significant dQTL included tree height **(A)**, MVD **(B)**, cMVD **(C)**, cVF **(D)**, VGI **(E)**, and bark thickness **(F)**. Genomic bin position is plotted against the −log10 of the value of *p* for each correlation test. A significance threshold corresponding to a value of *p* of 0.05 is depicted as a blue horizontal line. The values of *p* were adjusted using a relaxed Bonferroni correction (see section “Materials and Methods”).

**Figure 7:**
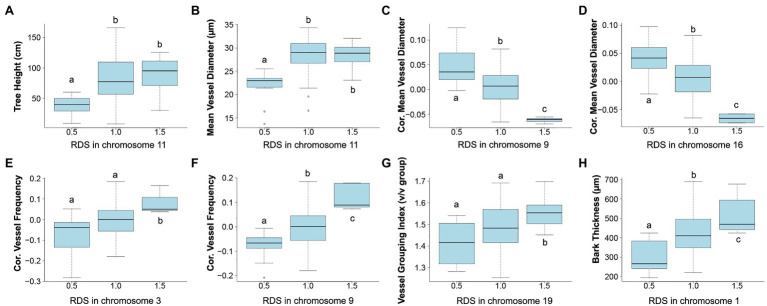
Relationship between trait value and relative dosage scores (RDSs) at genomic bins detected by dQTL analyses. Each boxplot shows all genotypes grouped by RDS at the most significant dQTL detected for each trait. Selected dQTLs were located on chromosome 11 for tree height **(A)** and MVD **(B)**, chromosome 9 **(C)** and chromosome 16 **(D)** for cMVD, chromosome 3 **(E)** and chromosome 9 **(F)** for cVF, chromosome 19 for VGI **(G)**, and chromosome 1 for bark thickness **(H)**. Tukey pairwise comparisons were used to visualize significant differences in mean trait values between the groups; means that share a letter are not significantly different (*p* > 0.05).

Correlations of dosage at genomic bins and each trait are shown in Figure 6 for TH, MVD, cMVD, cVF, VGI, and BT, and statistics for each dQTL bin including the percentage of phenotypic variance explained are summarized in Table 4. There were no significant correlations between the remaining traits (VF, MVC, and NF) and specific areas of the genome (not shown). Boxplots showing the relationship among dosage and trait values for each dQTL are shown in Figure 7. MVD and tree height were both significantly correlated with three contiguous genomic bins on chromosome 11, spanning 0.9 Mb (Figures 6A,B; Table 4). Lines with insertions in these regions did not significantly differ in MVDs or tree heights from lines lacking indels, while lines with deletions had significantly lower values for these traits (Figures 7A,B), potentially uncovering a maternal allele with unique function or lack of function. These dQTL explained 4.1% of MVD variance and 3.4% of tree height variance (Table 4) and contained 63 annotated genes. cMVD was significantly correlated to seven genomic bins in chromosome 9, and five genomic bins on chromosome 16 (Figure 6). Together, these bins covered a length of 5.6 Mb and accounted for 14.9% of the total phenotypic variance of the trait (Table 4). The dQTL within chromosome nine included 380 annotated genes, while the dQTL within chromosome 16 included 370 annotated genes. Lines with deletions spanning either the chromosome 9 (Figure 7C) or the chromosome 16 dQTL (Figure 7D) showed increased cMVDs, while lines with insertions showed the opposite phenotype, as might be expected for a dosage-sensitive negative regulator of growth. cVF was significantly correlated to five genomic bins in chromosome 9 and one genomic bin in chromosome 3 (Figure 6). These dQTL jointly covered 2.1 Mb that contained 246 annotated genes and explained 11.2% of the total phenotypic variance of the trait. cVF was not statistically significantly different in lines carrying deletions in the chromosome 3 cVF dQTL region compared to lines lacking indels (Figure 7E), while lines with deletions of the chromosome 9 cVF region did significantly differ from lines lacking indels (Figure 7F). Lines carrying insertions covering either the chromosome 3 or 9 cVF dQTL regions exhibited significantly higher cVF values (Figures 7E,F). The five dQTL bins on chromosome 9 associated with cVF overlapped with five most significant bins correlated to cMVD, consistent with the idea of a common regulator affecting both traits. VGI was significantly correlated to six genomic bins spanning 4.2 Mb within chromosome 19 (Figure 6) that was not implicated in dQTL for any other trait. Along with the observation that VGI did not show significant correlations with other traits (Figure 5), these results suggest that this dQTL may identify a genetic factor specific to this trait. VGI for lines with deletions in the chromosome 19 VGI dQTL region did not statistically differ from lines lacking indels in this region, while lines with insertions had statistically greater VGI (Figure 7G). This dQTL explained 4.2% of the trait variance and contained 379 annotated genes. Finally, BT showed significant dQTL bins spanning a large region of chromosome 1 (Figure 7H), which explained 7.2% of the trait variance. Gene dosage in this region was positively correlated with BT and was not implicated in dQTL for other traits, despite correlations of BT phenotype with tree height, MVD, and VF (Figure 5).

**Table 4:** Locations of all genomic bins with significant correlations (*p* < 0.05) with stem or wood anatomical traits (dQTL).

Trait	Chr	Location (MBP)	*p*	Variance explained (%)
TH	11	6.10–6.65	0.0338	3.4
TH	11	6.60–6.75	0.0361	
TH	11	6.75–7.00	0.0429	
MVD	11	6.10–6.65	0.0297	4.1
MVD	11	6.60–6.75	0.0222	
MVD	11	6.75–7.00	0.0416	
cMVD	09	5.40–5.60	0.0147	14.9
cMVD	09	5.60–6.30	0.0046	
cMVD	09	6.30–6.80	0.0004	
cMVD	09	6.80–6.90	0.0012	
cMVD	09	6.90–7.10	0.0032	
cMVD	09	7.10–7.50	0.0100	
cMVD	09	8.50–9.90	0.0481	
cMVD	16	0–0.50	0.0038	
cMVD	16	0.50–0.80	0.0047	
cMVD	16	0.80–1.00	0.0178	
cMVD	16	1.00–1.30	0.0150	
cMVD	16	1.40–2.20	0.0478	
cVF	03	4.60–5.30	0.0383	11.2
cVF	09	6.30–6.80	0.0072	
cVF	09	6.80–6.90	0.0039	
cVF	09	6.90–7.10	0.0032	
cVF	09	7.10–7.50	0.0078	
cVF	09	7.50–7.70	0.0358	
VGI	19	7.20–8.10	0.0283	4.2
VGI	19	8.10–9.10	0.0215	
VGI	19	9.10–9.60	0.0230	
VGI	19	10.6–10.8	0.0301	
VGI	19	10.8–12.2	0.0257	
VGI	19	12.2–12.4	0.0256	
BT	01	34.9–36.8	0.0273	7.2
BT	01	39.1–41.8	0.0488	
BT	01	41.8–41.9	0.0116	
BT	01	49.3–49.6	0.0405	

The adjusted *R*-squared of the linear regression model fitting the genomic bins was used to estimate the percentage of trait variance explained by dQTL.

## Discussion

The regulation of vessel element morphological traits remains poorly understood, despite the key role of vessel traits in determining the hydraulic physiology of plants, and trees in particular. One fundamental feature of vessel elements that makes their study complex is their extreme developmental plasticity. Vessel elements show dramatic morphogenic variation, as seen by contrasting vessel elements with narrow lumens and spiral secondary cell wall thickenings produced during elongative primary growth, and vessels with wide lumens and extensive secondary cell walls produced during secondary growth. Vessel element diameter and frequency have also been shown to be positively correlated with tree size (Olson and Rosell, 2013; Olson et al., 2014). And vessel development and final morphology are highly responsive to environmental conditions and can be modified to produce morphologies better suited to fast growth under permissive conditions or mitigating the effects of unfavorable conditions such as drought (Rodriguez-Zaccaro and Groover, 2019). A fundamental question is then, to what extent are vessel traits, such as final diameter, under genetic control, vs. non-genetic responses to environmental or physiological conditions?

Here, we took advantage of a genomically characterized population of *P. deltoides* x *nigra* to provide new insights into the genetic control of vessel traits associated with water transport physiology. This study is unusual as it presents trait distributions, correlations, heritabilities, and dQTL for wood anatomical traits directly in a forest tree species within a pedigree carrying indels that create additional, dosage-based variation. All the anatomical and vessel traits under study here showed significant heritabilities, indicating a significant degree of genetic control. As anticipated, some stem anatomical and vessel traits showed significant correlations with tree height. Specifically, significant positive correlations were found between tree height and MVD (0.76) and BT (0.52), while VF showed a significant negative correlation (−0.76) with height. One goal was thus to determine the amount of phenotypic variation in the MVD and VF is attributed to genetic variation not associated with height. We analyzed this by removing the effect of tree height in ANOVA to calculate cMVD and cVF that were “corrected” for the effect of tree height. This approach was effective and showed that genetic variation independent of tree height had significant heritabilities for cMVD (0.44) and cVF (0.32). Thus, in addition to scaling by tree height, we observed additional significant variation among genotypes for MVD and VF, suggesting that these are traits amenable to genetic manipulation and breeding independent of height or tree size.

Our irradiation hybrid germplasm also enabled analysis genome-wide scans for quantitative trait loci that are both responsive to gene dosage variation and that affect vessel traits. We found significant QTL for tree height, MVD, cMVD, cVF, VGI, and BT. Other traits, VF, MVC, and NF, did not reveal significant QTL, however. In each case, there are several types of genetic variation that would be transparent to our analysis here. These include genes that are involved in vessel trait regulation but that do not show dosage sensitivity, genes with segregating allelic variation, genes whose variation is masked by genetic redundancy or physiological compensation mechanisms, and genes with smaller effects. Regardless, some interesting conclusions can be reached about the genetic architecture and interplay of the traits under study here. For example, the correlation of tree height with MVD is reflected in a common dQTL on chromosome 11, perhaps representing a common regulator influencing each trait, or else a factor directly influencing one trait with indirect influence on the other. After correcting for tree height, significant genetic variation was still detected for cMVD, as reflected by a unique dQTL on chromosome 9. Interestingly, the cMVD trait was inversely proportional to dosage at this dQTL, predicting that a dosage-sensitive negative regulator of growth may be uncovered at this locus. Additionally, unique dQTL not shared by other traits were identified for cVF, VGI, and BT, suggesting potential trait-specific regulators at these loci. In addition to furthering our basic understanding of how these various wood anatomical traits are related to each other genetically, these results also provide insights into what traits may be independently targeted through breeding approaches. In this regard, an identification of dQTL for vessel traits that are not shared by tree height is encouraging for the ability to select or breed for trees through selection on both height growth and vessel traits.

Previous studies point to challenges in the genetic dissection of wood anatomy and vessel morphological traits. Quantitative genetic analyses of vessel traits in a *P. deltoides* mapping population identified a single QTL with the causative locus *ENLARGED VESSEL ELEMENT* (*EVE*) encoding a plasma membrane-localized protein affecting potassium uptake and presumably turgor in differentiating vessel elements (Ribeiro et al., 2020). Interestingly the EVE protein is not only specific to vessel elements but also found in fibers, but nonetheless is supportive of the notion that potassium plays a key role in vessel expansion. A large genome-wide association study in *Populus trichocarpa* identified a single QTL associated with intermediate wood vessel size, encoding a putative double-stranded RNA binding protein (Chhetri et al., 2020). Multi trait associations with vessel area phenotypes were identified for an additional six genes, including genes encoding a leucine-rich repeat containing protein and a serine–threonine protein kinase that might play signaling roles during vessel element differentiation. Interestingly, some vessel traits were correlated with the latitude of accessions included in the study, emphasizing that these are adaptive traits are under genetic control and selection. Notably, there is no overlap among the QTL found in our study and any of these previous studies. Along with the modest number of QTL identified in these studies, it seems reasonable to speculate that the high responsiveness of vessel traits to environmental variation make it especially challenging to measure genetic signal against a background of uncontrolled environmental variation.

Our results clearly demonstrate that vessel traits are under genetic control and that dosage-responsive QTL can be identified. While this is encouraging, we also see the opportunity to increase the power of detection and characterization of the genetic regulation of vessel traits. Notably, future studies could exploit automated phenotyping systems to minimize uncontrolled environmental variation, impose water stress treatments to examine vessel trait responses, and capture physiological data using image-based phenotyping to correlate with those responses. Similarly, high throughput histological phenotyping combined with machine learning-based approaches could provide more insightful and uniform means for analyzing wood anatomy and vessel features. A systems genomics strategy integrating vessel phenotypes, genomic locations in indels, and gene expression data could now be used to both summarize mechanisms and identify candidate genes for functional characterization, as we recently used for dissecting poplar leaf morphology (Bastiaanse et al., 2021).

The magnitude of problems associated with climate change effects on forests is large with drought and increasing temperatures resulting in water stress that has already resulted in forest declines and associated wildfire in large areas of temperate and boreal forests. While vessel anatomy and hydraulics is only part of the picture, gaining new fundamental insights into how trees respond to environmental variation is a part of the needed information for producing solutions for managing and conserving forests in decades to come.

## Data Availability Statement

The datasets presented in this study can be found in online repositories. The names of the repository/repositories and accession number(s) can be found at: https://www.ncbi.nlm.nih.gov/genbank/. The genome sequencing datasets used for this study for detecting and defining indels can be found in NCBI SRA database under accession number SRP040492 and BioProject ID PRJNA241273.

## Author Contributions

AG, FDRZ, and IH designed the study. FDRZ led the data collection and the data analysis with direction from AG. FDRZ drafted the manuscript. AG and IH edited the manuscript. All authors contributed to the article and approved the submitted version.

## Conflict of Interest

The authors declare that the research was conducted in the absence of any commercial or financial relationships that could be construed as a potential conflict of interest.

## Publisher’s Note

All claims expressed in this article are solely those of the authors and do not necessarily represent those of their affiliated organizations, or those of the publisher, the editors and the reviewers. Any product that may be evaluated in this article, or claim that may be made by its manufacturer, is not guaranteed or endorsed by the publisher.
